# Supplementary material for: Boosting Clear Cell Renal Carcinoma-Specific Drug Discovery Using a Deep Learning Algorithm and Single-Cell Analysis
Source: Int J Mol Sci. 2024 Apr 8;25(7):4134. doi: 10.3390/ijms25074134 (PMC11012314; doi:10.3390/ijms25074134)
Supplement: Supplementary file 1 [file ijms-25-04134-s001.zip › Supplementary Figures and Table.pdf]

Table S1. The parallel table of T cell clustering in UMAP and cell types identified by marker genes.

Cell Types Clustered in UMAP	Cell Types Annotated	Marker Genes
cc-CD8+ T1	Unknown	None
cc-CD8+ T2	CD8 Trm	GZMA,GZMB,GZMK,HLA-C,IFNGAMMA,PRF1
cc-CD8+ T3	Tnaive	CXCL13
cc-CD4+ T4	Tregs	FOXP3,IL2RA,TIGIT,TNFRSF18,TNFRSF4

Supplementary Figures:

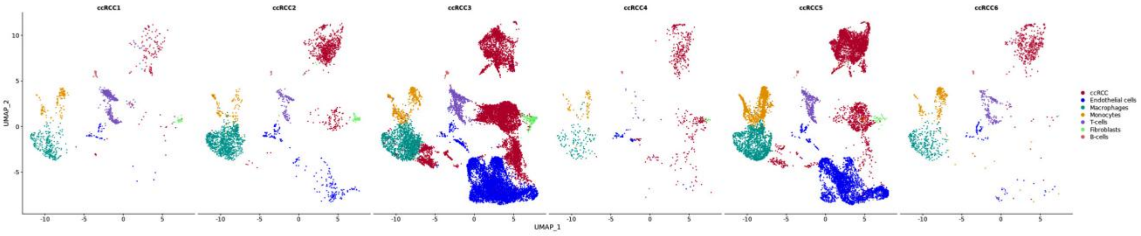

Figure S1. The distribution of different types of cells in six tumor samples.

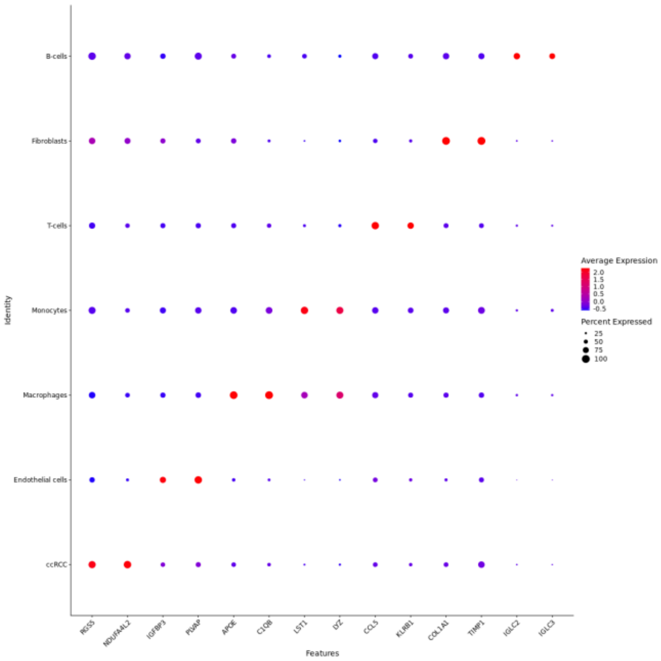

Figure S2. The expression levels of marker genes in different cell types.

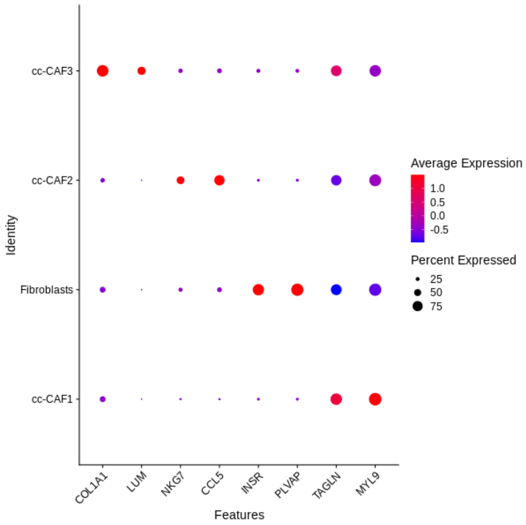

Figure S3. Differently expressed genes in different subtypes of CAF cells.

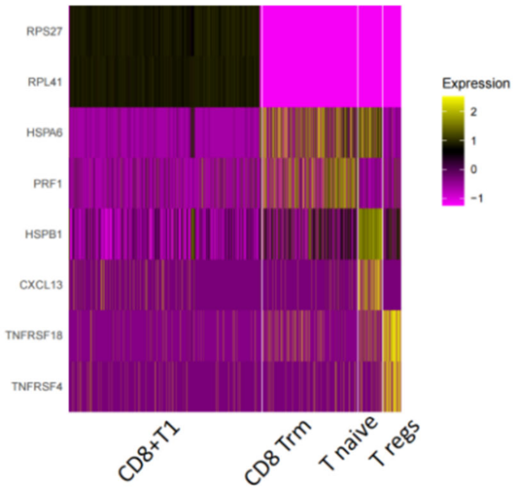

Figure S4. Gene expression characteristics of four types of T cells.

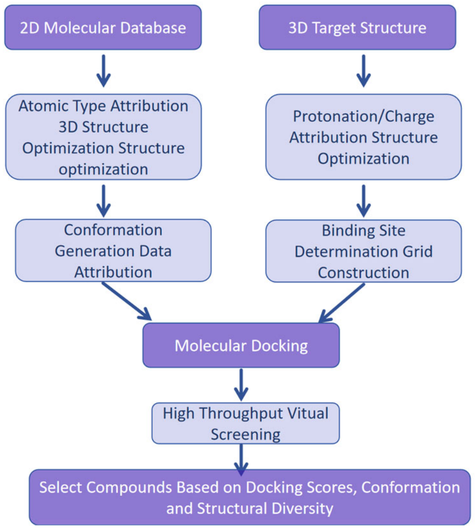

Figure S5. Workflow of the candidate drugs targeting EPAS1, selected via virtual screening.
